# Gut-lung axis, probiotics, and prebiotics: insights on dysbiosis, mechanism, and prevention of lung cancer

**DOI:** 10.3389/fnut.2025.1624803

**Published:** 2025-07-30

**Authors:** Deepika, Zayed D. Alsharari, Md Faruque Ahmad, Jagphool Singh, Mohini Yadav, Anita Kumari, Anil Kumar, Karl R. Matthews, Rotimi E. Aluko, António Raposo, Najla A. Albaridi, Boshra Mozaffar, Ariana Saraiva, Saurabh C. Saxena

**Affiliations:** ^1^Department of Nutrition Biology, Central University of Haryana, Mahendragarh, Haryana, India; ^2^Department of Clinical Nutrition, Faculty of Applied Medical Sciences, University of Tabuk, Tabuk, Saudi Arabia; ^3^Department of Clinical Nutrition, College of Nursing and Health Sciences, Jazan University, Jazan, Saudi Arabia; ^4^Centre for Medical Biotechnology, Maharshi Dayanand University, Rohtak, Haryana, India; ^5^Department of Biochemistry, School of Interdisciplinary and Applied Sciences, Central University of Haryana, Mahendragarh, Haryana, India; ^6^Department of Food Science, Rutgers University, New Brunswick, NJ, United States; ^7^Department of Food and Human Nutritional Sciences, University of Manitoba, Winnipeg, MB, Canada; ^8^CBIOS (Research Center for Biosciences and Health Technologies), ECTS (School of Health Sciences and Technologies), Lusófona University, Lisboa, Portugal; ^9^Department of Health Science, College of Health and Rehabilitation, Princess Nourah Bint Abdulrahman University, Riyadh, Saudi Arabia; ^10^Research in Veterinary Medicine (I-MVET), Faculty of Veterinary Medicine, Lisbon University Centre, Lusófona University, Lisboa, Portugal; ^11^Veterinary and Animal Research Centre (CECAV), Faculty of Veterinary Medicine, Lisbon University Centre, Lusófona University, Lisboa, Portugal

**Keywords:** gut-lung axis, lung cancer, microbiota, prebiotics, probiotics

## Abstract

**Background/objectives:**

Science continues to unravel the intricacies of the human body that affect health and well-being. The human gastrointestinal tract is inhabited by microbiota responsible for various bodily functions and reactions. This review provides an overview of lung-related diseases and the role of the gut-lung axis (GLA) related to lung cancer. Integral to the discussion are the roles that prebiotics and probiotics play in critical aspects of the GLA. The review aims to bridge the conventional knowledge with recent knowledge of therapeutic agents.

**Methods:**

A literature review was performed using the keywords “gut-lung axis,” “prebiotics,” “probiotics,” and “lung cancer” on Google Scholar, PubMed, and JSTOR.

**Results:**

Probiotic bacteria have a positive effect in maintaining beneficial bacteria in the gastrointestinal tract, which subsequently shows significant effects in maintaining lung health. Coupled with the efficacy of probiotics is a diet rich in prebiotics required to maintain a healthy gastrointestinal tract microbiota. Probiotics and prebiotics have specific mechanisms of action to prevent lung health-related diseases such as lung cancer. Recent advances have shown the potential of non-toxic and sustainable therapeutic agents for reversal of dysbiosis in GLA.

**Conclusion:**

Research suggests that the gastrointestinal tract microbiota has a crucial role in the onset and prevention of lung cancer. Lung cancer, which is prevalent in almost every country around the globe, is found to be associated with the occurrence of various other co-morbidities. Seeking new methods to maintain lung health and prevent lung diseases, including lung cancer, remains urgent, especially in the post-COVID era.

## 1 Introduction

Lung cancer accounted for 1,796,144 deaths in 2020, which is nearly 18% of all cancer deaths globally (1). The India National Cancer Registry Programme, linked with the Indian council of medical research estimated a significant increase in the age-adjusted incidence rate of lung cancer, irrespective of gender since the early 1980s. The GLOBOCAN 2018 report indicated lung cancer as the fourth leading type of cancer (5.9% cases) in India among all age groups and gender. Furthermore, 63,475 deaths (8.1% of total cancer related deaths) were due to lung cancer (cumulative risk 0.60), making it the third leading cause of cancer-related mortality (2). Further, the GLOBOCAN 2022 report indicated that lung cancer was the first most common cancer in males and the second most common cancer in females worldwide in 2022 accounting for 1.57 and 0.91 million new cases reported in males and females, respectively. Lung cancer was also reported as the most common cause of cancer deaths in the men (3). The long prognosis period for lung cancer and low 5-year survival rate affirms a terrifying situation (4). Another study indicates that 24,80,675 and 18,17,469 deaths occurred from lung cancer in 2022 worldwide. However, these numbers would elevate by 86.2 and 95.2% by 2050 (5). Lung cancer is attributed to various factors such as sedentary lifestyle, western dietary habits, alcohol consumption, smoking, environmental pollution, and microbial dysbiosis. Delayed identification of cases of lung cancer leads to a greater number of disease-related mortality.

Several studies in the past few decades have identified the role of microbes as a key determinant for the health spectrum of an individual. A large heterogenous population of microbes (fungi, viruses, bacteria, protozoans) inhabit the human gastrointestinal tract (GIT) and are linked with prevention and treatment of various disorders such as irritable bowel syndrome (IBS), Crohn’s disease, pulmonary infections, cancer, COVID-19 and neuropsychological diseases (6–8).

Beneficial bacteria in the GIT provide essential life functions such as metabolizing food, drugs, vitamins, and inhibiting pathogenic microbes; these commensal bacteria have astonishing effects against the onset and in the prevention of numerous illnesses including lung cancer. Pathogenic bacteria, which may enter the human body through abrasions or in ingested food contribute towards deterioration of health of an individual and this leads to the development of various disorders linked to GIT dysbiosis (i.e., an imbalance in the gut microbiota) (9). In the condition of gut dysbiosis, pathogen populations expand and release toxins having health deteriorating effects, which may result in cancer genesis, genomic instability (due to DNA breakdown and disturbance in DNA repair pathway), inflammation, virulence promotion, and cell cycle disruption (10). For example, Cag A protein, a protein synthesized by *Heliobacter pylori*, a gastrointestinal pathogen promotes the onset of cancer (11). Of the many responses that may occur with GIT dysbiosis, genotoxin production and inflammation are responsible for onset of lung cancer. Correcting GIT dysbiosis through reestablishing bacterial populations has a positive health influence and may result in reversal of harmful physiological effects. Probiotic and prebiotic interventions of non-small cell lung cancer (NSCLC), which accounts for up to 85% of lung cancer cases can lead to positive outcomes (12). The GIT microflora can be influenced by diet such as the Mediterranean diet, which positively effects GIT microbe growth and subsequently an individual’s health. Indeed, nutritional intervention using prebiotics and vitamins has become popular. For example, vitamin D deficiency has also been associated with increased risk of lung diseases and its administration is found associated with reduction in lung illnesses related mortality (13). Thus, research activities that are related to the impact of microbes on the gut-lung axis (GLA) and gut-brain axis (GBA) have expanded rapidly. Hence, considering the above information, this review was designed with an aim of discussing the GLA in detail alongside highlighting the possible dysbiosis that might occur in GLA which causes the lung cancer such as inflammation, altered responses of immune system, microbial dysbiosis, and epigenetic regulation. Further, the uniqueness of the review lies in its aims to highlight the possible options for managing the elevating cases of lung cancer owing to advancement in food science. This review highlights the importance of functional foods, i.e., probiotics and prebiotic administration for curbing the prevalence of lung cancer.

## 2 Gut-lung axis

Despite their physical separation, the respiratory and gastrointestinal systems have strong anatomical similarities and a shared embryonic origin, raising the prospect of multimodal interactions between these two systems. As a result, a novel and distinct relationship between the GIT and respiratory tract has been identified as the gut-lung axis (GLA). Of the recognized inter-organ interactions, the GLA still requires greater understanding than the others. Researchers suggested that the two-way regulation of the GLA involves microbial and immunological processes (14). It is an intricate communication that links the microorganisms in the digestive tract and lungs via blood and lymphatic circulatory systems. Renz et al. (15) reported that the intestinal flora can influence the lung flora through the circulatory system and contribute to a number of adverse respiratory conditions, such as chronic obstructive pulmonary disease (COPD), cystic fibrosis, and asthma (16). The complex connections between diverse elements of the gut and lungs’ microbiota and local and distant immunological responses lead to coining the phrase GLA in reference to the bidirectional process. The alteration or destabilization of the axis may have detrimental effects, including pathogen colonization, increased susceptibility to infections, tissue damage, and cancer development (17). Initial steps in colonization of the gut and lungs are comparable, with both having strong mucosal barriers against microbes. Notable similarities exist between the pulmonary and intestinal mucosae. For instance, goblet cells of the intestinal mucosa may release IgA while concurrently producing IgA in the respiratory mucosa. Moreover, the lungs and intestines can influence one another’s immunity (18). Previous studies have shown that short-chain fatty acids (SCFAs), the primary metabolic by-products of dietary fat produced by the gut microbiota, can regulate lung immunity, and mediate immunological functions of the gut microbiome in an allergy paradigm (19, 20). Additionally, the pulmonary immune response is also regulated by bacterial lipopolysaccharide and immune cells such as the TREG, which impact microbes that populate the lungs (21).

## 3 Drivers of the lung–gut interactions

**Figure 1:**
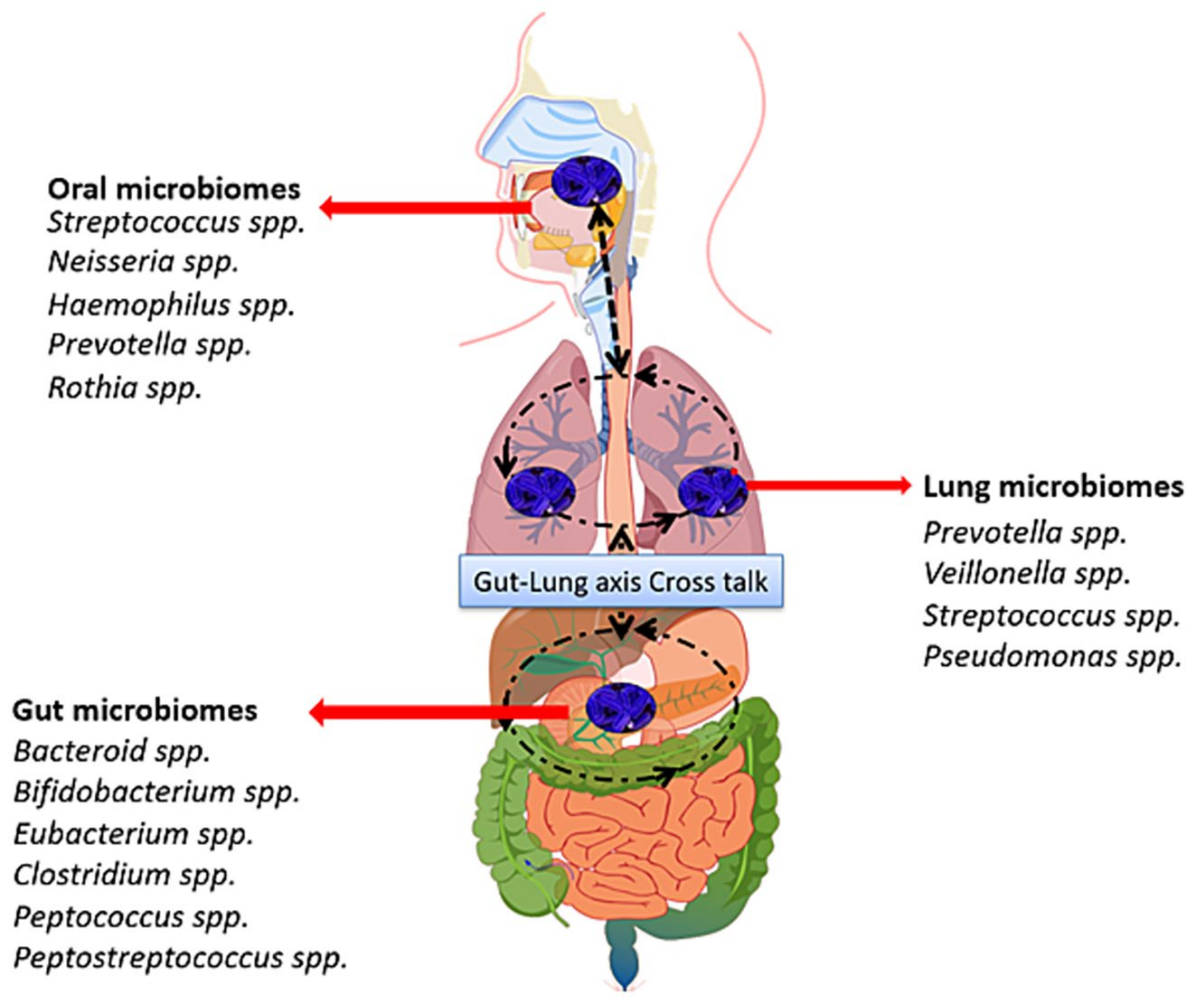
Interactive communications between lung, gut and oral microbiomes: The oral, pulmonary, and intestinal microbiomes can interact directly through mucosal dispersion, respiratory, and digestive functions, and indirectly through inflammatory chemicals, cytokines, and metabolites that circulate in a regular pattern. Intestinal bacteria and their metabolites control the likelihood of native T cells to differentiate and the release of Th17, which controls systemic inflammatory response and immunity.

Being a dynamically balanced system, the human body offers various sites for the interactions of microorganisms via direct (mucous dispersal, digestive and respiratory activities) and indirect (cytokines, inflammatory substances and circulating metabolites release) (Figure 1). The microbiome community structure of the lung is primarily influenced by three factors, i.e., growth rate, migration, and elimination of microbes under normal and pathological conditions (22, 23). A few studies have suggested that the oral microbiome contributes primarily to the lung microbiome by swallowing of mucus and micro-secretion released in oral cavities (22). Biological processes such as micro-aspiration and inhalation also function in communication between the digestive tract and the respiratory system (24). In the GIT, the temperature and pH generally remain constant, and migration of microbes is unidirectional and influenced by a variety of chemical and physical factors. In contrast, the lung frequently exchanges gasses with the surrounding environment to retain oxygen levels and the microbiome. Additionally, limitations of a physical barrier and variability in temperature and pressure allows the bidirectional migration and dynamic alteration of the lung microbiome (25). The in-silico analysis using clusters of orthologous groups of proteins (COG) and Kyoto Encyclopedia of Genes and Genomes (KEGG) databases have suggested that gut dysbiosis can alter the metabolic processes and may have a connection with lung cancer (26). Energy consumption has been considered as a significant element in the etiology of cancer; however, improved immune response and homeostasis depends on the gut microbiota. The latter may release various bioactive compounds that can harm the host, by altering the metabolism of carbohydrates including starch, fructose, sucrose, pentose, galactose, glucuronate, mannose and ribose. Further, the conversion of undigested carbohydrates and proteins into acetic acid provides an ultimate source of energy for the microorganisms, including firmicutes.

Based on histopathological findings, researchers have identified gut microbiome characteristics in lung cancer; however, these correlations are less well described and require further elucidation. Furthermore, carcinogenesis and tumor formation leading to cancers could be influenced by the multiple and diverse modes of dysbiosis. Despite the involvement in many metabolic processes, the gut microbiome exhibited less involvement in the pathways related to energy metabolism and ABC (ATP-binding cassette)-type transport in patients having lung cancer (27). This indicates an intricate connection between the gut bacteria and the host; strengthening the theory that lung cancer is a metabolic disorder. Further, the *Bifidobacterium*-derived extracellular vesicles (*Bif*.BEVs) have also been quoted as influential factor for immunotherapy response in condition of NSCLC. *Bif*.BEVs are internalized by lung cancer cells primarily through dynamin-dependent endocytosis. Thus, in addition to microbial metabolites, extracellular vesicles (EVs) derived from the gut microbiota may also play a crucial role as a driver for interaction of gut-lung axis (28). Additionally, the microbiota may interfere with physiological homeostasis resulting from neurological and cognitive dysfunction, immunological dysregulation, and perturbed metabolism of the host.

## 4 Dysbiosis and lung cancer

Lung cancer has emerged as a significant health risk to humans in recent decades based on the high morbidity and mortality rates associated with the disease. Research indicates that the use of certain antibiotics, such as penicillin, macrolides, and cephalosporins, is associated with an increased risk of developing lung cancer (29). These antibiotics have the ability to alter the gut flora, which points to the possibility of a connection between lung cancer and the gut microbiome. The term “dysbiosis” refers to an imbalance or disruption in the composition and function of the microbiota of the host. One of the illnesses that has been linked to dysbiosis is lung cancer. Contrary to common belief, a low-density, diverse microbial ecosystem has been discovered to reside in the lungs (30). Recent findings suggest that lung cancer may be linked to an imbalance in the microbiota of the lungs at multiple stages of the disease’s progression. A dysbiotic lung microbiota may influence metabolic processes, in addition to immune and inflammatory responses. All these factors can aid in tumor growth and metastasis (30). Several studies have shown that people with lung cancer experience shifts in the composition of their lung microbiota. These studies show that there are numerous mechanisms that could explain why dysbiosis is linked to lung cancer (31, 32). The dysbiotic composition of the lung microbiota has been linked to an increased risk of developing lung cancer. Potential mechanisms of DNA damage include increased mutation load, alterations in inflammatory responses, abnormal activation of signaling pathways and production of multiple cytokines and bacterial toxins (32). Additionally, cytokines, toxins, and other pathways produced by dysbiotic lung microbiota may disrupt lung homeostasis and promote tumor growth (32). Direct and indirect interactions between the microbiota of the lungs and other microbial communities in the body, such as the microbiota of the mouth and the gastrointestinal tract, allow for communication between these disparate systems (32). The potential impacts resulting from dysbiosis at the gut-lung axis have been discussed below.

### 4.1 Altered immune response

A significant percentage of the macrophage population has been observed in the submucosal layer of the digestive tract and mesenteric lymph nodes. Moreover, gut microbes also express pathogen-associated recognition receptors (PARR) and pathogen-associated molecular patterns (PAMPs), e.g., segments of lipopolysaccharides (LPS) that can be recognized by PARR on immune cells (21). The protein or cell wall portion of living or dead microbes enters the mesenteric lymphatic nodes and subsequently into the intestinal system, thereby evading encountering cytokines and chemokines. If the first line of defense does not eliminate microbial ligands, they reach the lung via the bloodstream and trigger the toll-like receptor (TLR) innate-adaptive immune response resulting in the activation and differentiation of immune cell types. Additionally, commensal bacteria produce metabolites like SCFAs (butyrate and propionate) that directly stimulate intestinal epithelial cells and control the release of immune cells (33). Researchers introduced the concept of cancer-immunity cycle to explain the significance of the gut microbiota in the anticancer response. They suggested that dendritic cells’ (DCs) acquisition of neoantigens initiates the cancer-immunity cycle. However, to have an efficient immune response, release of proinflammatory cytokines and other elements is required. The DCs present the neoantigens to T cells, that allow their activation and priming to become effector T cells that help to fight against cancer-specific antigens. The ratio of effector to regulatory T cells is crucial for determining the type of immune response. The activated effector T cells move to tumor locations, cause infiltration of the tumor bed, and bind to tumor antigens to facilitate cancer cells eradication. However, under certain conditions, DCs and T lymphocytes recognize antigens as “self” rather than as alien, resulting in regulatory T cells (Treg) response instead of effector response. It has been previously known that commensal microbiota has the ability to stimulate the CD4 + T-lymphocytes development against self-antigens (34, 35). Zitvogel et al. (36) proposed the two signal hypotheses to explain the cross-reactivity and anti-tumor surveillance concept. The first hypothesis suggests that specific microbial proteins mimicking the tumor antigens penetrate the intestinal barrier and evoke the immune response via cross-reactivity. The second hypothesis suggests that commensal microbiota by interacting with PRRs may drive production of cytokines and interferons production that elicit the immune responses. The microbial translocation and alteration of intestinal barrier functioning is thought to influence interplay between the gut microbiome, immune system, and diseases, including lung cancer (16).

### 4.2 Inflammation

The gut-lung axis is vital for determining the effect of lung infections on the blood microbiome and intestinal microbiota; however, the underlying mechanism depends on the lung inflammation impact on the pathological reactions in intestinal tissue. Inflammation is linked to the movement of microorganisms from the gastrointestinal system into the bloodstream (37). Kim et al. (38) reported that lung inflammation elicits systemic innate immune response that may increase the vulnerability of the intestine to inflammatory effects. Various gut microbes including Bacteroides, Propionibacterium, Bifidobacterium, Eubacterium, Lactobacillus, Roseburia, Clostridium, and Prevotella are excellent producers of SCFAs (39). The release of SCFAs from dietary fibre and its intestinal absorption by gut microbes may improve resistance to lung infection by lowering the infiltration of inflammatory cells and airway inflammation (19). The most significant SCFA is butyrate, produced mostly by members of the Firmicutes. Butyrate exhibits a variety of anti-inflammatory activities, including activation, adhesion, proliferation and migration of immune cells, cytokine expression, and apoptosis of cancer cells. Additionally, it can serve as the primary energy source for the intestinal epithelium and play a vital role in maintaining the barrier integrity (21, 40, 41). Butyrate is thought to inhibit the activity of histone deacetylase (HDAC) that affects the proinflammatory cytokines secretion profile, tumor cell proliferation and apoptosis (42–44). In addition to diet, a variety of factors, including antibiotics, chemotherapy, and an individual’s overall immunological status may also alter the gut microbiota, resulting in dysbiosis with a temporary or long-lasting effect (21). Moreover, a lack of the proper composition at the beginning of the immune response has more significant repercussions than just the immediate pathogenic effects. Considering all the factors, it is understandable that a “healthy” microbiota is vital for the fundamental development of the immune system.

### 4.3 Altered metabolism

A wide range of bioactive compounds that bacteria produce can influence the metabolic processes of their hosts. For instance, deoxycholic acid and lithocholic acid, which are produced from bile acids by gut bacteria and have the potential to cause damage to DNA and are thought to play an important part in the early stages of cancer (45). In addition, carcinogenic metabolites like acetaldehyde and deoxycholic acid have been found linked with the etiology of liver and esophageal cancers. The findings from a few studies suggest that an improperly functioning metabolism can lead to production of toxic metabolites, which in turn may promote the progression of lung cancer (19, 46). Research has been carried out in order to demonstrate the role of bacterial metabolites in the development of lung cancer. For instance, it has been demonstrated that the human lung adenocarcinoma A549 cell line is susceptible to the apoptosis-inducing agent cytolethal distending toxin (CDT). CDT is a bacterial genotoxin that is produced by a variety of Gram-negative bacteria, including Actinobacillus (47). In addition, *G. adiacens* has been associated with the development of lung cancer. Surprisingly, high concentrations of polyamines such as putrescine and gamma aminobutyric acid have been linked to a variety of diseases including lung cancer (48). Apopa et al. (49) observed an increased abundance of cyanobacteria in lung carcinoma after treating lung adenocarcinoma (A427) cell lines with microcystin. The outcome of functional analysis also demonstrated that the toxin (microcystin) produced by Cyanobacteria could boost the procyclic acidic repetitive protein 1 (PARP1) levels, which would subsequently increase inflammation and ultimately lead to cancer.

### 4.4 Virulence and genotoxicity

The disruption and modification of bacterial genome can be genotoxic resulting in production of a variety of toxins, as well as free radicals, DNA lesions, an arrest in the cell cycle, and apoptosis without the possibility of DNA repair. Therefore, destabilization of the microbiome can have a carcinogenic effect on the host organism (50). DNA damage is a well-known and important factor in the development of cancer. Genotoxins cause damage to the DNA of host cells through one of two distinct mechanisms: either they form adducts or they cause double-stranded breaks. Double-stranded breaks can lead to mutations, insertions, deletions, or chromosomal inversions and translocations if they are not repaired by the host cell’s natural DNA repair systems. The CDT, which is produced by proteobacteria, is another agent that can cause damage to DNA in a similar fashion (51). The metabolites produced by gut microorganisms are capable of producing free radicals and having an effect on reactive oxygen species (ROS); as a result, these metabolites have the potential to also have an indirect genotoxic effect. In addition, studies have demonstrated that bile acids rapidly produce reactive nitrogen species (RNS) and ROS, both of which are capable of causing DNA damage in the host cell (52). Alterations in the microbiota composition can cause ROS levels to rise, which in turn induce DNA damage and carcinogenesis. Research demonstrates that bacterial toxins like cytotoxic necrotizing factor 1, CDT, and the *Bacteroides fragilis* toxin are capable of mediating damage responses in double-stranded DNA (47, 53–56). Additionally, it was observed that free radicals such as superoxide and hydrogen sulfide, which are both produced by bacteria, are the root causes of chromosomal instability (57).

### 4.5 Dysbiosis of microbiota

Lung cancer (LC) is one of the most lethal forms of cancer, and there are two subtypes of it: small-cell lung cancer (SCLC) and non-small-cell lung cancer (NSCLC). SCLC is more common than the NSCLC (58). To improve patient survival and therapeutic outcomes, it is essential to gain a better understanding of the mechanisms through which the microbiome may influence the progression of lung cancer cases. It is hypothesized that there is a complex multifactorial interaction between the human microbiome and lung cancer; however, the microbiota of the lungs has not been studied as thoroughly as those of the gastrointestinal tract (59). Antibiotic use may alter the population and configuration of the microbiota, the ratio of commensal to pathogenic microbiota, and the risk of developing lung cancer (29). Therefore, a dysbiosis or imbalance in the microbiota is strongly associated with the incidence of lung cancer. In addition, imbalance in the microbiota and pathogenic bacterial flora, plays a key role in the development of lung cancer by increasing the production of cytotoxic agents and inflammatory mediators.

Patients with LC are typically infected with pathogens belonging to the Firmicutes, Proteobacteria, and Bacteroidetes superfamilies (60). These superfamilies include taxa such as Streptococcus, Granulicitella, Mycoplasma, and Veillonella. It has been demonstrated that LC frequently harbors Gram-negative bacteria, including Enterobacter spp., *Escherichia coli*, and *Haemophilus influenza* (61). Considering the gut microbiota composition, it is essential to keep in mind that patients with LC have lower concentrations of Firmicutes and Proteobacteria when compared to healthy people, in addition to relatively increased levels of Bacteroidetes and Fusobacteria (62). It appears that these phyla are always present, despite the fact that cancer associated microbes may change.

Chronic infection of the lungs may be the first step towards the development of cancer when microbial dysbiosis produces an environment that is more hypoxic and promotes the growth of tumors. Furthermore, the bacteria that preferentially colonize tumors possess elective anaerobic characteristics that lead to a rise in the rate of anaerobic respiration, which can be observed in LC. As the LC progresses, these bacteria proliferate, further contributing to an atmosphere within the tumor that is deficient in oxygen and promotes inflammation. In addition to the effect that cancer treatment has on tumors, a growing body of evidence suggests that it can also affect harmful microbiota (63). Changes in one tissue impact the other because of the GLA’s bidirectional connection between the GIT and the lungs. The passage of gut microbiota and their by-products through the epithelial barrier and into the bloodstream is an important regulatory mechanism (16). Additionally, translocation sets off a response mediated by toll-like receptor (TLRs), which results in the proliferation of T cells in far-flung areas (64). The translocation of bacteria from the GIT can boost tumor-specific responses through TLRs or induce memory responses, as is seen in the relationship between *Enterococcus hirae* and SCLC (65).

### 4.6 Epigenetics regulation

The microbiome constitutes between 1 and 3% of our body weight. Numerous microbes can alter or disrupt human genes (66, 67). There is more protein-coding DNA in a person’s microbiome than in their actual genome. Recent research has uncovered a large number of microproteins encoded by diverse organisms (68). Another major contributor to cancer progression is epigenetic dysregulation, which has a particularly profound effect on lung cancer. DNA methylation, chromatin modification, and non-coding RNAs are all examples of epigenetic regulatory processes that can lead to shifts in gene expression. One of the most common mechanisms to silent the genes is DNA methylation, which modifies the chromatin structure by adding a methyl group (69, 70). CpG island methylation, which is frequently observed in cancer cells, silences the associated gene. Histone proteins, which enclose the DNA, undergo numerous post-translational modifications (PTM) such as methylation, acetylation, phosphorylation, ubiquitination, glycosylation, and ADP-ribosylation. These modifications to histones ultimately determine how genes are activated or silenced via distinct transcriptional mechanisms. Non-coding and regulatory RNAs add a new layer of complexity to the epigenetic regulation of genes (71).

Since epigenetic modifications can be passed down from generation to generation, it is important to learn how environmental cues like microbial dysbiosis affect epigenetic memory and transcriptional homeostasis in order to gain a more mechanistic understanding of diseases like cancer. Bacterial infections cause changes to their hosts’ epigenomes, which they use to perpetuate their population, reproduce, and evade the innate immune defenses of their hosts. TLR4 activation by bacterial lipopolysaccharide (LPS) results in the nuclear translocation of NF-kB, which then activates several inflammatory genes. These early response genes have a high transcription rate because of their epigenetic and transcriptional positioning. However, further signaling and chromatin modifications are required for the late response genes to become active. In order to colonize and reproduce, both pathogenic and commensal bacteria can alter the host’s chromatin landscape. High levels of commensal bacteria are found in areas of the human body that regulate inflammatory responses. To achieve this fate, chromatin remodeling could be triggered at inflammatory gene loci (72).

The epigenetic modifications that are brought by the microbiome in the host are the result of unknown molecular processes. The epigenome can be altered by a wide variety of microbial metabolites, including butyrate, folate, and biotin (73). Butyrate is a SCFA that is produced when bacteria ferment food fibers; it has a strong HDAC inhibitory action. It controls many different functions within the host organism (72, 74, 75). Folate is produced by many bacteria, including Lactobacillus and Bifidobacteria, and then converted into 6-methyltetrahydrofolate, a methyl group donor that affects DNA methylation (76). HDAC3 is induced by the microbial metabolite phytate via. Inositol triphosphate, and this aids in intestinal homeostasis and repair (77). Biotin, a bacterial compound, is essential for the ongoing biotinylation of proteins, especially histones. Biotinylation of histones H3, H4, and H2A regulates many cellular functions, including DNA repair, cell cycle arrest, and gene silencing (78, 79) as it opens chromatin to different transcriptional factors. Histone acetylation and deacetylation are crucial mechanisms for regulating transcription. Bacteria are the primary source of the acetyl group, which is required to make acetyl-CoA, a donor for histone acetylation (73).

**Figure 2:**
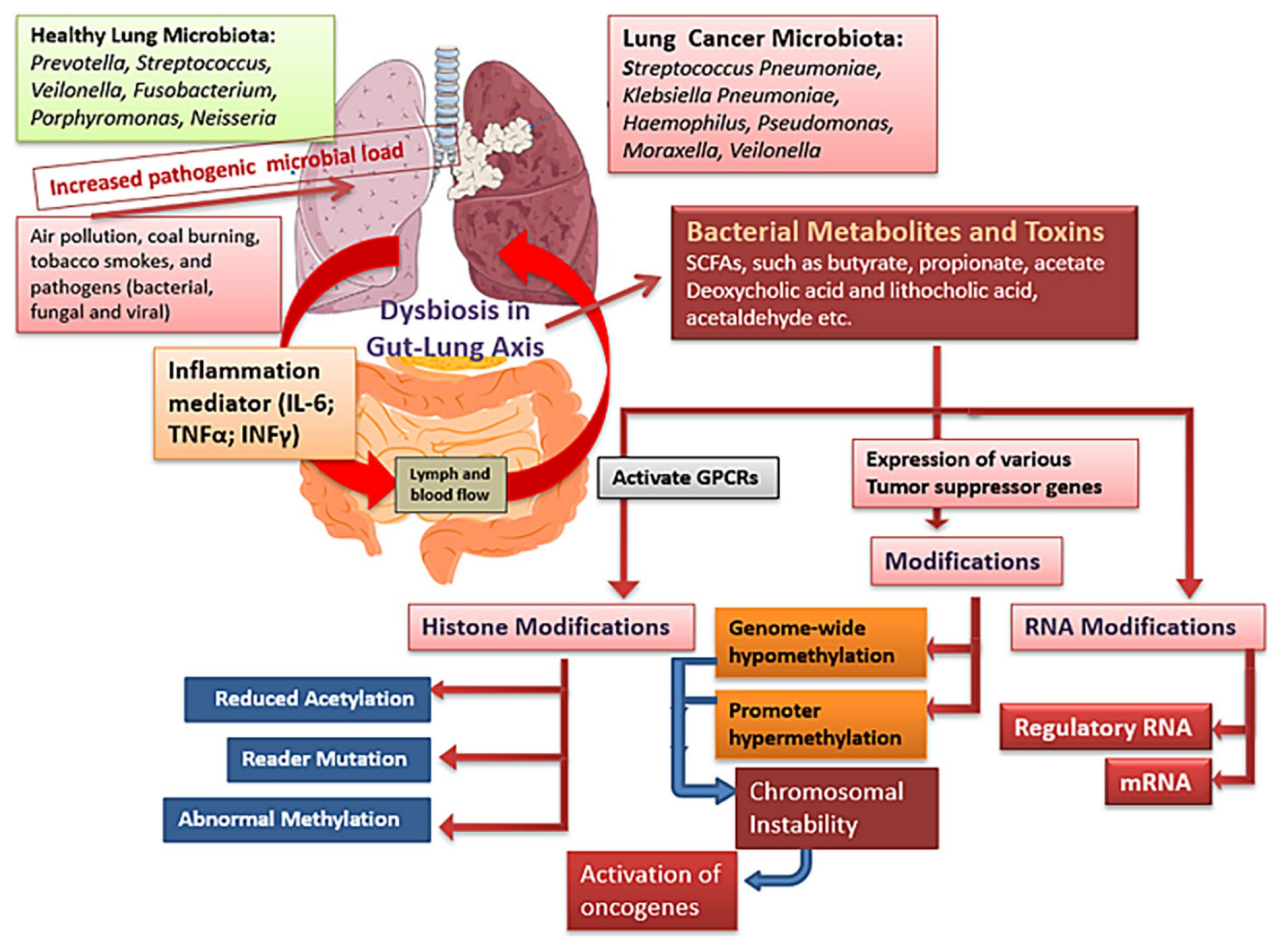
Impact of dysbiosis in gut-lung axis: Many factors such as air pollution, tobacco smoke, burning coal, and other pollutants constantly expose the lungs to inflammation, which can affect microbial dysbiosis. Alterations in the microbial community of lungs and persistent lung inflammation can accelerate cancer development.

The microbiome of the lungs is less well understood than the microbiome of the digestive system. As a result, extensive mechanistic research is being carried out to understand their role in regulating the host epigenome to promote lung carcinogenesis. However, microbial dysbiosis in LC has been studied extensively, and it has been known that microbial metabolites have the ability to influence the epigenome of the host (Figure 2). When it comes to controlling the immune responses of the lungs, nothing is more important than the HDAC inhibiting activity of SCFAs, which is mediated by the gut-lung axis (80–83). These findings suggest that dysbiosis along the gut-lung axis can suppress antitumor immune responses and promote lung carcinogenesis. Both pathogenic and commensal *Streptococcus pneumoniae* reside in the lungs, and for this microbe to invade and produce pneumolysin toxins, the pyruvate oxidase enzyme must dephosphorylate histone H3 at serine position 10 via the host’s PP1 phosphatase (113). The correlation between increased H3S10 phosphorylation and carcinogenesis suggests that commensal Streptococcus spp. may play a vital role in maintaining H3S10 phosphorylation homeostasis in the lungs (84). It is well understood that the microbiome has considerable impact on the host’s epigenetics. However, in order to successfully prevent lung cancer, it is important to understand how lung microbiome dysbiosis affects the host’s ability to maintain epigenetic homeostasis (85).

## 5 Intervention with probiotics as treatment agents against lung cancer

The viable microbes inducing positive health benefits through the gut associated axis are a topic of intense research interest nowadays. The world health organization (WHO) states probiotics are “live/viable microbes which when ingested in a certain amount confers health benefits to the host.” The role of probiotics in modulating adverse conditions of LC has been highlighted in several studies. A strong relationship exists between probiotics, oncogenesis, and anti-cancer impacts. The precise mechanism(s) behind the theory has not been elucidated, however, according to some studies, the probiotic intervention as a diet source has a positive impact on the health condition of an individual suffering from respiratory and lung diseases such as LC. For example, bioactive compounds found in Kefir, a fermented drink made from kafir grains (a symbiotic association of yeast and bacteria) and milk, has a significant inhibitory effect on induction and proliferation of tumor cells (86). Similarly, regular consumption of other probiotic dietary foods such as yoghurt have a positive influence against various health disorders including LC (87). The probiotics may be bacterial strains such as those belonging to genera *Lactobacillus and Bifidobacterium*; or non-pathogenic yeasts such as *Saccharomyces boulardii*. The regimen of chemotherapy for LC treatment leads to adverse effects on the body, which may include damage to the intestinal mucosal layer and other adverse effects on the GIT. Research suggests probiotic intervention can have remarkable results against carcinogenesis (88). The administration of *Lactobacillus rhamnoses GG*, and *Bifidobacterium animalis* subsp. *lactis Bb12,* led to promising effects against cancer in patients (8). Gastrointestinal microbiota (in terms of richness or dysbiosis) is identified as an essential factor for response against cancer immunotherapy (89). In certain LC cases like NSCLC, the property of metastasis out of lungs are a cause of concern among researchers and health experts as they cannot be removed surgically (4). Recent literature (Table 1) accounts for studies showing significant relationship between administration of probiotic/prebiotic and condition of cancer and related disorders. The mentioned studies have concluded that intestinal-pulmonary balance of microbiota via direct administration or dietary oral consumption will prolong the survival rate and enhance the quality of lives of patients suffering from lung diseases with specific reference to LC. The process of immune-tumor-editing during the condition of LC can be done through these biologically active therapeutic agents in three phases or 3 e’s; elimination, equilibrium, and escape (90). This way, the microbes will act as an important gateway for better therapeutic outcomes in patients suffering from lung cancer. These microbes may either act as an immunomodulatory agent or activators for defense pathways (91). Although the underlying mechanisms are poorly understood, immunotherapy outcomes seem to depend on host-related factors like gut microbiome alpha diversity, relative abundance of microbial genera/taxa, and extrinsic factors such as prior or concurrent exposure to probiotics, antibiotics, and other microbiome-modifying drugs. The gut and lung microbiota are a dynamic microecosystem and the composition of same will define the condition inside the body of a host (92). Besides prevention and treatment against lung cancer and its related negative impacts in the body, these health ameliorating microbes will also be beneficial to fight against pathogenic microbes, which flourish in the host during bacterial dysbiosis. Pathogenic microbes belong to genera *Streptococcus, Veillonella*, and *Mycoplasm*a. Some Gram-negative bacteria namely *Haemophilus influenzae, Enterobacter* spp., and *Escherichia coli* also flourish in the case of bacterial dysbiosis during LC (59). Additionally, the therapeutic efficacy of impaired response to immune check point blockade due to antibiotics is also found associated with gut microbiota and probiotic intervention (93). Therefore, probiotics or probiotics enriched food sources influence carcinogenesis and immunotherapy in LC and associated diseases (94). Probiotics will certainly act as future drugs or novel therapeutics against the health deterioration and invasive illness conditions (95).

**Table 1:** Studies showing significant relationships between administration of probiotic/prebiotic and condition of cancer and related disorders.

S. No.	Probiotic/prebiotic administered	Conditions	Study model	Results	Reference
1	*Bifidobacterium longum*	Colon tumor incidence	Male F344 rats	suppress the expression of ras-p21 oncoprotein activity, ornithine decarboxylase activity, and cell proliferation induced by azoxymethane	(9)
2	Kefir (mixture of yeast and bacteria)	Tumor	–	inducing apoptosis and preventing tumor cell proliferation	(86)
3	*Enterococcus hirae and Barnesiella intestinihominis*	Cancer	Antibiotic treated mice	Activate Th1 and Tc1 anticancer responses induced by CTX and promote IFN-γ + *γ*δT cell infiltration into cancerous lesions	(108)
4	*Lactobacillus acidophilus*	Lung cancer	Lewis lung cancer mouse model	Reduce the amount of IFN-γ, GZMB, and PRF1 that CD8 + T cells produce while increasing the expression of VEGFA and downregulating the expression of BAX and CDKN1B	(109)
5	Fructo-oligosaccharide	Adenomas	Human	Increase in amount of butyrate.	(101)
6	Inulin	Melanoma tumor	Mice	Promote antitumor immunity, Triggered greater infiltration of immune cells (CD45^+^, dendritic cells)	(99)
7	Inulin	Tumor	Mice	Triggers potent alpha beta T cell anti-tumor immunity	(99)
8	Oligofructose	Mammary tumors	Rats	Anticarcinogenic effect of oligofructose	(110)
9	Inulin or Oligofructose	Liver tumor and mammary carcinoma	Mouse	Tumor growth inhibitory effects.	(111)
10	Inulin or oligofructose	Lung cancer	Mice	Anti metastatic effect	(112)

## 6 Intervention with prebiotics as treatment agents against lung cancer

In the past, prebiotics were referred to as “non-digestible food components that positively affect recipient by enhancing development of colonic bacteria that are currently available” (96). Prebiotics are precursors, which are exploited by bacteria to improve host health (97). They promote microbial species’ development in the gut to boost host’s health. Prebiotics are compounds that enter the colon in their whole, that is, without being damaged by the digestive acids and stomach pH. Prebiotics also serve as nutrients for colonic probiotic bacteria by promoting their development. Generally, prebiotics encourage development of *Bifidobacteria* and *Lactobacilli* over pathogenic bacteria. Most prebiotics are oligosaccharides with short chains, which are composed of 3–10 units of carbohydrates and are obtained from various plants. Based on their chemical structures, prebiotics have mostly been divided into 2 classes: galacto-oligosaccharides and inulin-type fructans (98). Inulin, a soluble dietary fiber that is resilient to gut enzymes and hence enters the large intestine, is one example of an indigestible but fermentable dietary carbohydrate that specifically stimulates the development of colon microbes. A diet rich in inulin not only stimulates strong antitumor immunity in T cells but also, in a microbiota-dependent manner, leads to the accumulation and activation of intra-tumoral T cells. A diet high in inulin significantly changes the microbiota’s composition and, predictably, encourages the growth of *Bifidobacterium* species, which are known to stimulate the immune system. It promotes antitumor immunity and triggers greater infiltration of immune cells (CD45^+,^ dendritic cells) in mice (99).

Galacto-oligosaccharides enter the colon undigested, where they traverse the bowel and encourage the development of microbes that are regarded to be healthy (100). Studies shows that when compared to a control group of rats fed a basal diet containing starch as the only carbohydrate, the addition of 15% oligosaccharides to a rat diet decreased the incidence of tumors, thereby negatively modulating rat mammary carcinogenesis induced by methylnitrosourea. Oligosaccharides show anti carcinogenic effect on mammary tumors of rats. In a study patients with gynecological cancer were given the dose of 50% inulin and 50% fructo-oligosaccharide, twice daily from 1 week before to 3 weeks after radiotherapy. Every day, the quantity and quality of the stools were noted. Prebiotics contribute to more regular stools in patients getting radiation therapy for gynecologic cancer. Studies assessing the involvement of inulin-type oligofructose in cancer have primarily examined colorectal cancer. According to animal studies, oligofructose and inulin reduce tumor growth, act as anti-carcinogenic and anti-metastatic agents, and enhance the effects of cancer therapy for colorectal cancer. The actions of inulin and oligofructose in inhibiting the development of cancer cells are related to an increase in *Bifidobacteria* in the colon and the cell wall preparations they produce. Additionally, the two prebiotics’ proliferative and apoptotic properties are linked to a reduction in glucose availability, which is a crucial substrate for cancer cells (101). Many foods and their constituents that meet the following criteria have been identified as having prebiotic characteristics:

1. Refractory to being digested and absorbed by the host.
2. Fermented by the GIT microflora.
3. Slightly increases one or a small number of bacteria’s activity or development within the digestive tract.

Integrative medicine often recommends healthy meals full of raw and cooked vegetables, lean protein, omega-3 polyunsaturated fatty acids, vitamin D3, and probiotics and prebiotics. This mix of meals and the right supplements may significantly reduce inflammation in the body and intestine (102).

The bacteria of the genera *Bifidobacterium* and *Lactobacillus* that limit the presence of pathogenic bacteria, are most supplemented with prebiotics to promote health (103). They include oligofructose, inulin and lactose. It was discovered that the activities of genotoxic enzymes reduce when prebiotics are administered (102). A study on effects of feeding galacto-oligosaccharides to people revealed a drop in the putrefaction markers indole and isovaleric acid, that are formed by deamination and protein breakdown (103). High levels of cecal butyrate helps in prevention of cancer by acting as a primary source of energy for colonocytes and by maintaining a healthy epithelium. Moreover, it can be very helpful in preventing cancer. These interactions include apoptosis activation, which would typically contribute to death and a rise in immunogenicity of cancer cells because of increased protein expression on the cell surface. Further, recent studies in this regard have discussed the potential of food-derived extracellular vesicles (FEVs) for cancer prevention. FEVs are promising prebiotics for treatment of various diseases, including lung and liver cancer. FEVs can withstand severe degradation conditions of the gastrointestinal tract. This special characteristic makes FEVs a promising prebiotic in nutritional and health studies. These FEVs can be isolated from plant, milk, or probiotics (104). Moreover, exosomes generated from plants provide a non-toxic source of anti-cancer drug development, resolving concerns regarding patient health and impact to environment. Thus, plant derived exosomes lays down a new niche for exploring prebiotics from various sources as potential therapeutic agent owing to the offered efficiency and sustainability (105).

**Figure 3:**
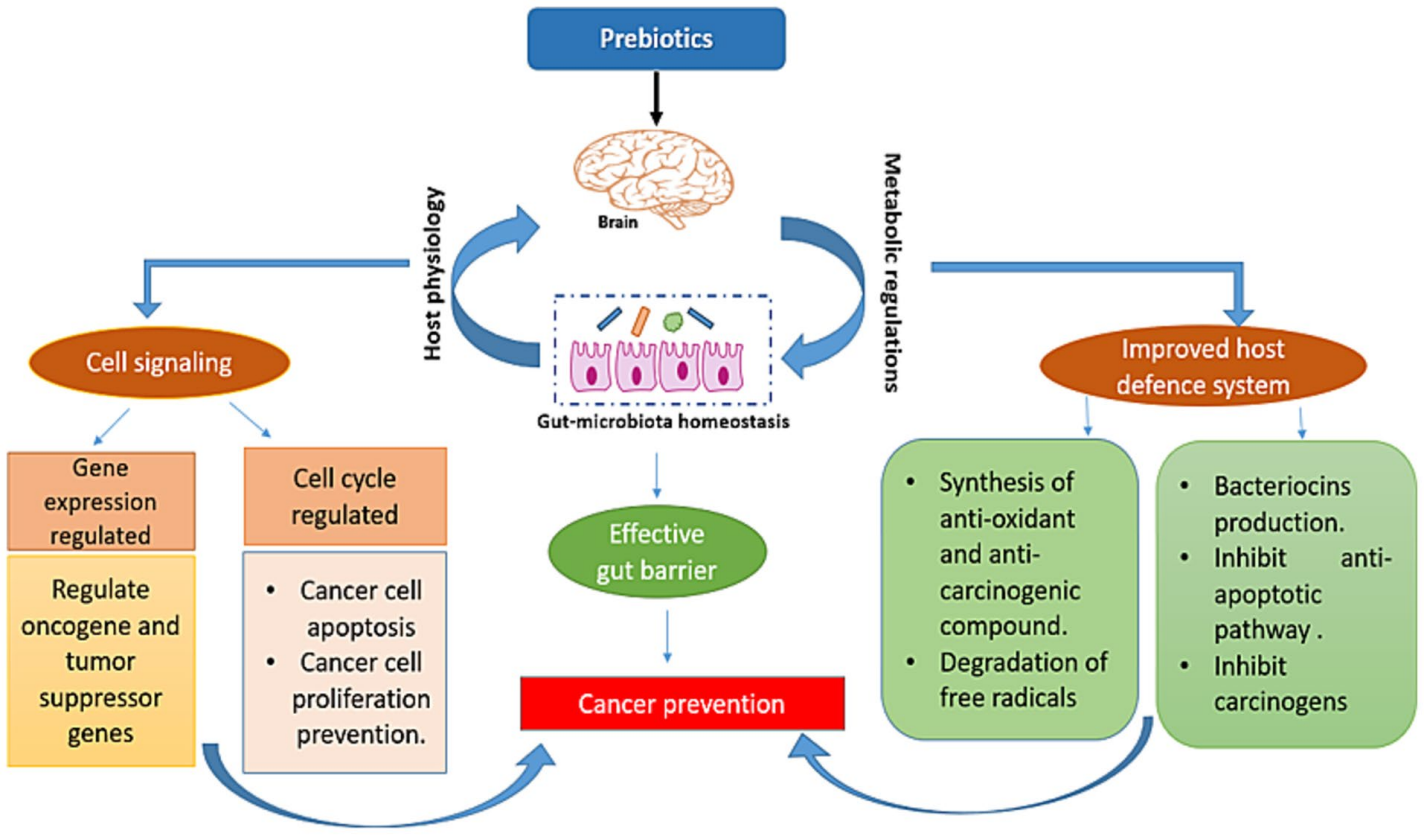
Mechanism of cancer prevention by prebiotics.

When probiotics digest prebiotics, SCFAs are created in the colon, which have anticancer effects on the host through a number of mechanisms, such as altering metabolic activity of gut microbes, enhancing intestinal health, altering immunological function, binding and degrading carcinogenic substances, altering tumor gene expression, altering the differentiation processes in tumor cells, having antimutagenic effects, altering the host’s physiology, and inhibiting cell proliferation. The postulated mechanism of cancer prevention by prebiotics is shown in Figure 3.

## 7 What’s ahead?

The study of commensal lung microbiome populations and potential mechanisms for microecological impacts on the human respiratory system has attracted more research. Research is required to determine the function of gut microbiota in onset and development of LC, as well as to examine and assess the possible effects of the microbiome on the efficiency of anticancer therapy modulation (106). Future therapeutic advancements are expected to be significantly aided by nutraceuticals, but their success will depend on how they balance innovation with purity, safety, and efficacy. Since they complement today’s lifestyle, nutraceuticals will continue to be in demand. However, there is currently no well-defined approach to directly exclude abnormalities among lung cancer patients and healthy individuals in order to exclusively link LC to microbial regulation. The aforementioned innovations and more extensive medical studies will aid in the development of a better secure and reliable microbial LC therapy systems.

Key areas:

- Using a combination of probiotics intervention and chemotherapy holds promise in reducing the prevalence of gastrointestinal problems and avoiding degeneration of gut microbiota in LC patients.
- Additional biological effects provided by probiotics include the detoxification of carcinogens, reduction of cholesterol levels, improvement of lactose intolerance, synthesis of active metabolites including organic acids, and enhancement of vitamin production (90).
- Ability of certain probiotic bacteria and their metabolites to serve as therapeutic agents against cancer opens whole new avenues for the study and management of LC.
- Oncogenesis and tumor development may be impacted by changes in the microbiome on many different levels (90).
- Molecular modification of current traits of certain probiotic strains and creation of innovative engineered probiotic organisms focused on specialized qualities will enhance the treatment and prevention of different malignancies.
- The need to elevate the level of related studies in terms of clinical trials is certain. Majority of the findings in concern to this topic are associated with animal model-based studies. For applicability of any research finding, it is necessary to have abundant scientific proof to ensure its authenticity. Hence, the need of clinical trials is evident to ensure the dose specificity, gender specificity, bioavailability, bioaccessibility, and effects of these therapeutic agents in humans for specific disease.
- Innovative therapeutic strategies such as probiotics and prebiotics supplementation have been used to tackle side-effects of chemo-and radiotherapy. However, they might not be always beneficial. According to a systematic review, 85% of included studies showed health amelioration from probiotic supplementation; however, 15% of included studies did not show any positive impact on variables upon probiotic supplementation (107). To the best of our knowledge no study could be spotted wherein the limitations or side-effects of supplementing the probiotics in oncology patients or models were recorded. However, this does not remove the concern of any possible side-effects of these therapeutic strategies until all the key questions are addressed with proven research.

## 8 Conclusion

Research has established a strong link that gut microbes are directly implicated in cancer biology, including tumor development and responsiveness to anticancer medicines. Probiotics are made up of a range of microorganisms, such as bacteria and yeasts. Probiotics are crucial to human health since they produce several antimicrobial compounds. The literature suggests that gut-microbes have an impact on immunomodulation, decreased inflammation, and the restoration of gut homeostasis. Potentially, probiotics may be utilized as an adjuvant treatment in conjunction with anticancer medications that lower inflammation. Probiotics have also proven to be a highly successful therapeutic alternative for the treatment and prevention of gastrointestinal illnesses, urogenital infections, and dental caries. Prebiotics, on the other hand, are categorized as non-digestible dietary components that favorably encourage the development and metabolism of beneficial bacteria in the intestines, enhancing gut health of the host. Probiotics and prebiotics have an essential role in affecting microbiota, which in turn affects immune system development and disruption. Probiotics and prebiotics also work in harmony with the host to maintain a balanced and effective immune response, which further protects against pathogen colonization. The regular use of probiotics and prebiotics may enhance and modify the immune system by controlling production of anti-inflammatory cytokines and the genes that control their production. Dietary supplements that include an appropriate blend of prebiotics and probiotics improve the host’s health. Selection of appropriate prebiotics and probiotics may act as a preventative measure against LC development.
